# Endothelial dysfunction in small arteries and early signs  of atherosclerosis in ApoE knockout rats

**DOI:** 10.1038/s41598-020-72338-3

**Published:** 2020-09-17

**Authors:** Simin Berenji Ardestani, Ingrid Eftedal, Michael Pedersen, Per Bendix Jeppesen, Rikke Nørregaard, Vladimir V. Matchkov

**Affiliations:** 1 Department of Clinical Medicine, Aarhus University, Palle Juul Jensens Boulevard 99, 8200 Aarhus N, Denmark; 2 Department of Circulation and Medical Imaging, Faculty of Medicine and Health Sciences, NTNU Norwegian University of Science and Technology, Trondheim, Norway; 3 Faculty of Nursing and Health Sciences, Nord University, Bodø, Norway; 4 Department of Biomedicine, MEMBRANES, Health, Aarhus University, Aarhus, Denmark

**Keywords:** Cardiovascular diseases, Atherosclerosis

## Abstract

Endothelial dysfunction is recognized as a major contributor to atherosclerosis and has been suggested to be evident far before plaque formation. Endothelial dysfunction in small resistance arteries has been suggested to initiate long before changes in conduit arteries. In this study, we address early changes in endothelial function of atherosclerosis prone rats. Male ApoE knockout (KO) rats (11- to 13-weeks-old) were subjected to either a Western or standard diet. The diet intervention continued for a period of 20–24 weeks. Endothelial function of pulmonary and mesenteric arteries was examined in vitro using an isometric myograph. We found that Western diet decreased the contribution of cyclooxygenase (COX) to control the vascular tone of both pulmonary and mesenteric arteries. These changes were associated with early stage atherosclerosis and elevated level of plasma total cholesterol, LDL and triglyceride in ApoE KO rats. Chondroid-transformed smooth muscle cells, calcifications, macrophages accumulation and foam cells were also observed in the aortic arch from ApoE KO rats fed Western diet. The ApoE KO rats are a new model to study endothelial dysfunction during the earlier stages of atherosclerosis and could help us improve preclinical drug development.

## Introduction

Cardiovascular disease is the leading cause of death globally^1^. Endothelial dysfunction is known to play a central role in the progression of cardiovascular disease and changes in endothelial function are reported to occur prior to the development of atherosclerotic plaques^2–4^.

Atherosclerosis is a chronic inflammatory disease^5–7^. In its early phases, it is associated with dyslipidemia, increased expression of inflammatory factors and endothelial dysfunction^8,9^. At later stages, atherosclerotic lesions develop in conduit arteries^9^. The aorta and its major branches constitute the classical sites of atherosclerotic lesions. Endothelial dysfunction in small resistance arteries has been suggested to initiate long before changes in conduit arteries^10–12^. Therefore, understanding the mechanisms that trigger early endothelial dysfunction in resistance arteries are essential for our understanding of the early stages of atherosclerosis pathology, and consequently, early diagnosis and prevention. However, there are few reports of atherosclerotic plaques in other arteries such as the pulmonary artery^13,14^.

Laboratory animals are widely used to study endothelial dysfunction in cardiovascular disease, however, none of the current models reproduce human pathology in detail and there is no optimal model^15^. It has been shown that dyslipidemia itself does not cause atherosclerotic changes in rats^16^. Mice and rats do not normally develop atherosclerosis, but genetic modifications have been shown to facilitate its development^15^. Apolipoprotein knockout (ApoE KO) mice have been widely used to study atherosclerosis pathology and treatment^15^. ApoE plays an important role in cholesterol transport and metabolism by facilitating the removal of very low density lipoprotein (VLDL) remnants^17^. Elevated VLDL, LDL and triglycerides cause dyslipidemia, which is linked to development of oxidative stress and atherosclerosis^18^. However, experimental atherosclerosis development in mice is different from that in humans. In contrast to ApoE KO mice, which rapidly develop atherosclerosis when fed a high-fat diet, humans develop atherosclerosis slowly over the course of months or years^15,19^. With the development of genome editing technology, ApoE KO rats are now available and several studies have showed that atherosclerotic lesions develop slower in rats than in mice^20,21^. There is an ongoing discussion about the amount of lesion development in ApoE KO rats, and it appears that the location of targeted genomic change might influence the development of lesions^22,23^. To date, there are no comprehensive reports that characterise the vascular properties of ApoE KO rats.

Therefore, in this study, we examined the vascular phenotype of small (so-called, resistance) pulmonary and mesenteric arteries in ApoE KO rats fed a high-fat Western diet. Our aim was to assess the development of endothelial dysfunction in these small arteries and the mechanisms that promote atherosclerosis development.

## Results

The body weight was similar in all groups before starting the diet intervention at the age of 11–13- weeks-old. At the end of the study, at the age of 31–37 weeks; body weight increased significantly in all groups; (661.0 ± 55.40 g) in ApoE KO on Western diet, (586.30 ± 67.69 g) in ApoE KO on standard diet and (574.0 ± 10.77 g) in the Sprague Dawley rats on standard diet (*P* < 0.0001). The increase in weight was greater in ApoE KO on a Western diet and there was a significant difference in body weight between this group and ApoE KO and Sprague Dawley rats on a standard diet (*P* = 0.0005).

### Plasma total cholesterol, LDL and triglyceride was elevated in ApoE KO rats fed on a Western diet

**Figure 1:**
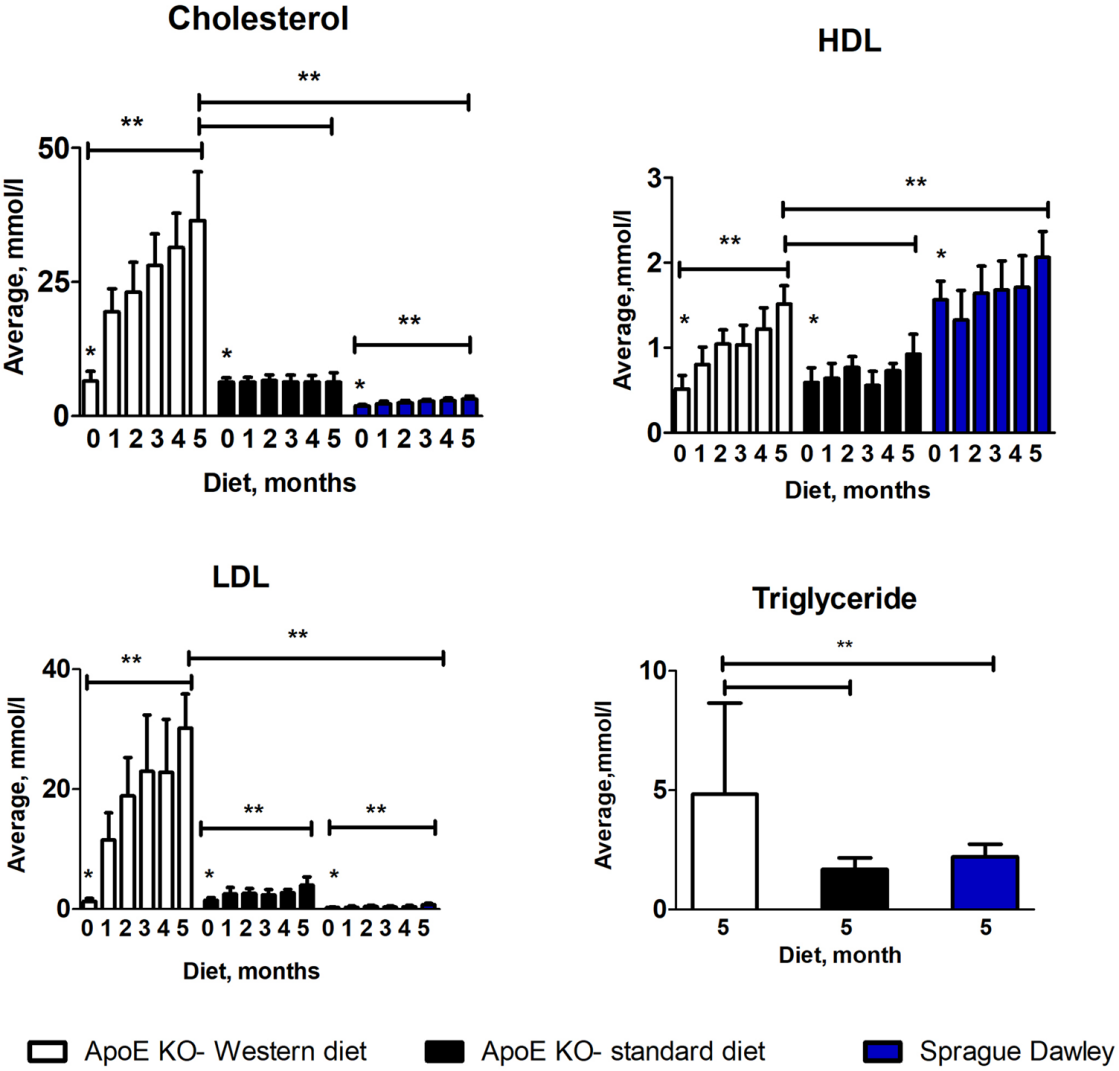
Monthly plasma cholesterol, LDL and HDL level and fasted triglyceride level vs diets in ApoE KO and Sprague Dawley rats. Cholesterol and LDL levels were significantly higher before the start of diet in ApoE KO rats compared to the Sprague Dawleys rats, (**) *P* < 0.0001; After 5 months of diet, cholesterol, LDL and triglyceride levels were significantly higher in ApoE KO rats fed on western diet compared to the ApoE KO and Sprague Dawley rats on standard diet, (**) *P* < 0.0001; HDL level was significantly higher in Sprague Dawley rats before and after control diet compared to the ApoE KO rats, (**) *P* < 0.0001; ANOVA, n = 8, n = 7.

The plasma cholesterol level was significantly higher in ApoE KOs compared to Sprague Dawley rats before the onset of the different diets (Fig. 1; Table 1, *P* < 0.0001, *n* = 15). After 5 months of diet, plasma cholesterol was significantly increased in ApoE KO rats on Western diet and Sprague Dawley rats on standard diet but not in ApoE KOs on standard diet. The effect was striking for ApoE KO rats on the Western diet, where cholesterol levels increased > ten-fold compared to Sprague Dawley rats on standard diet (Fig. 1; Table 1, *P* < 0.0001, *n* = 8). For ApoE KOs on standard diet, the cholesterol level was still doubled compared to Sprague Dawley rats on standard diet (Fig. 1; Table 1, *P* = 0.0004, *n* = 7).

**Table 1:** Summary of lipoprotein levels over time.

	Total cholesterol (mmol/L)		
	ApoE KO western diet	ApoE KO standard diet	Sprague Dawley standard diet
Baseline	6.53 ± 1.75	6.32 ± 0.79	1.89 ± 0.24
Following 5 months diet	36.43 ± 9.10	6.30 ± 1.80	3.14 ± 0.54
**LDL (mmol/L)**			
Baseline	1.30 ± 0.47	1.44 ± 0.46	0.23 ± 0.07
Following 5 months diet	30.20 ± 5.69	3.94 ± 1.42	0.71 ± 0.22
**Triglyceride (mmol/L)**			
Following 5 months diet	4.82 ± 3.83	1.68 ± 0.47	2.20 ± 0.53
**HDL (mmol/L)**			
Baseline	0.51 ± 0.15	0.59 ± 0.17	1.56 ± 0.22
Following 5 months diet	1.51 ± 0.21	0.92 ± 0.23	2.06 ± 0.30

Data presented as mean ± SD; ApoE KO: ApoE knockout.

Before diet commencement, ApoE KOs had significantly higher plasma LDL levels compared to Sprague Dawley rats (Fig. 1; Table 1, *P* < 0.0001). Plasma LDL increased significantly in ApoE KOs after 5 months on either Western (Fig. 1; Table 1, *P* < 0.0001, *n* = 8) or standard diet (Fig. 1; Table 1, *P* = 0.0009, *n* = 7). Plasma LDL also significantly increased in Sprague Dawley rats on standard diet (Fig. 1; Table 1, *n* = 8). However, the plasma LDL level was significantly higher in ApoE KO rats on Western diet compared to ApoE KO and Sprague Dawley rats on standard diet (Fig. 1; Table 1, *P* < 0.0001).

Triglyceride levels were measured after 5 months of diet; levels were significantly higher in ApoE KOs on Western diet compared to ApoE KO and Sprague Dawley rats on standard diet (Fig. 1; Table 1, *P* < 0.0001).

On the other hand, plasma HDL levels were significantly higher in Sprague Dawley rats compared to ApoE KOs before the diet started (Fig. 1; Table 1, *P* < 0.0001). Plasma HDL levels significantly increased in all groups after 5 months of diet, however, it was still significantly higher in Sprague Dawley rats (Fig. 1; Table 1, *n* = 8) compared to the ApoE KOs on Western (Fig. 1; Table 1, *P* < 0.0001, *n* = 8) or standard diet (Fig. 1; Table1, *P* = 0.01, *n* = 7).

### Immunohistochemistry

**Figure 2:**
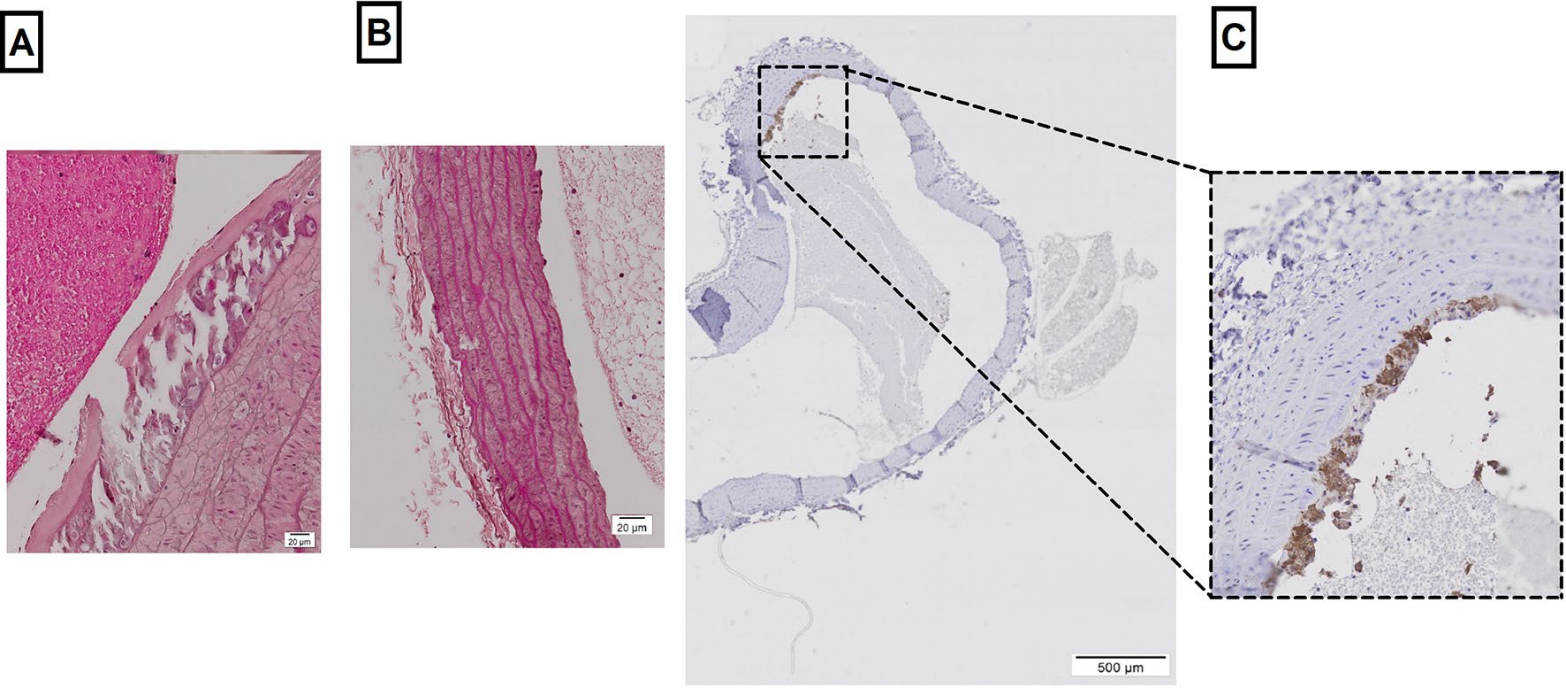
Microscopic images of the aortic arch from ApoE KO rat fed Western diet; (**A**) H & E staining at 60 × magnification showed chondroid-transformed smooth muscle cells; (**B**) H & E staining in Sprague Dawley rat; (**C**) CD-68 staining revealed accumulation of macrophages and small foam cell lesions.

The results from H & E staining showed chondroid-transformed smooth muscle cells and calcifications in ApoE KO rats fed on the Western diet, which revealed common features of atherosclerotic lesions (Fig. 2A). No changes were observed in Sprague Dawley rats (Fig. 2B). Further staining with CD-68 revealed accumulation of macrophages and small foam cell lesions only in ApoE KO fed Western diet (Fig. 2C).

### COX-dependent signaling was modified in pulmonary arteries from ApoE KO rats fed on a Western diet

**Figure 3:**
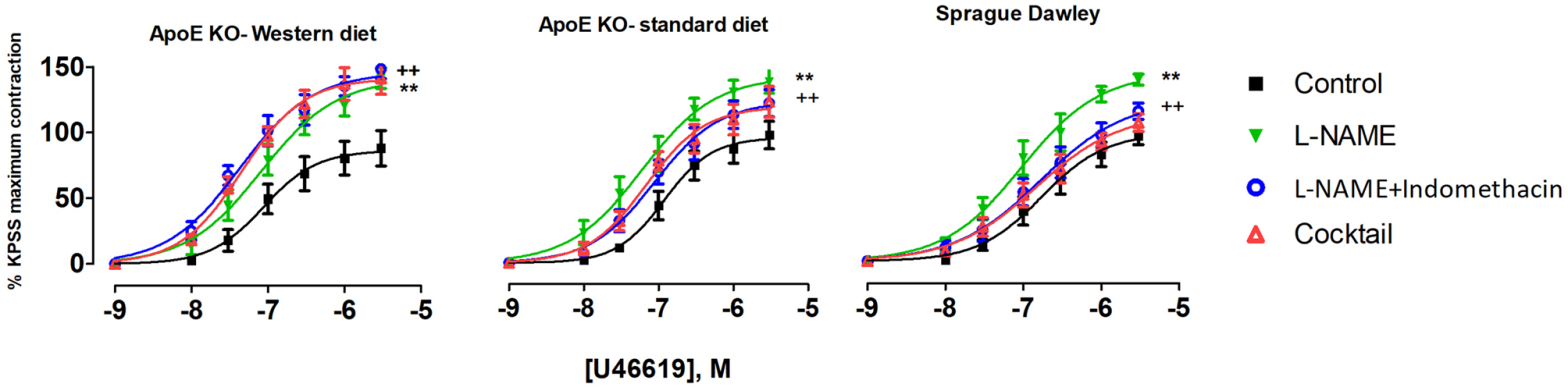
U46619 concentration–response curves of pulmonary arteries from different experimental groups under different experimental conditions; control conditions, incubation for 20 min with non-selective inhibitor of NO synthase, L-NAME (100 µM), incubation for 20 min with both L-NAME (100 µM) and indomethacin (3 µM); 20 min incubation with Cocktail: L-NAME, indomethacin, TRAM-34 and apamin, i.e. pharmacological inhibition of all major pathways for endothelium-dependent relaxation; (**), P < 0.001, effect of L-NAME; (+ +), P = 0.007, effect of indomethacin, F test, n = 8, 7.

Vessel contraction was expressed relative to the maximal contraction of KPSS (100% of contraction). There were no differences in contractile responses to KPSS between the experimental groups (Supplementary fig. S3A). Contractile responses of pulmonary arteries to increasing concentrations of the thromboxane A_2_ receptor agonist, U46619, were similar in all groups under control conditions. (Fig. 3).

The contraction response was significantly potentiated in all experimental groups in the presence of L-NAME (Fig. 3; *P* < 0.0001). The difference between the contractile response was not different between the groups (Supplementary fig. S4B).

In pulmonary arteries of ApoE KO-Western diet rats, contractile responses to U46619 were significantly potentiated by pre-incubation with both L-NAME and indomethacin (Fig. 3, Table 2, *P* = 0.01). However, pre-incubation with L-NAME and indomethacin reduced the contractile responses to U46619 in ApoE KO-standard diet and Sprague Dawley rats (Fig. 3, Table 2, *P* = 0.007 and 0.002, respectively). In the presence of indomethacin, the U46619 contractions were significantly different between ApoE KO-Western diet and either ApoE KO or Sprague Dawley rats on standard diet (Supplementary fig. S4C, *P* < 0.0001), suggesting that net COX activity in pulmonary arteries from ApoE KO-Western diet rats contributed to production of vasorelaxing factors while it had pro-contractile action in arteries from ApoE KO-standard diet or Sprague Dawley rats.

**Table 2:** Summary of logEC50 ± SE in pulmonary arteries in response to U46619 and mesenteric arteries to ACh in control situation; 20 min incubation  with L-NAME; 20 min  incubation with LNAME and indomethacin; 20 min incubation with with cocktail: L-NAME, indomethacin, TRAM-34 and apamin; E_max_, maximum contractile response; R_max_, maximum relaxation response.

Incubation with	ApoE KO-western diet		ApoE KO- standard diet		Sprague Dawley	
	*n* = 8		*n* = 7		*n* = 8	
	−logEC50 ± SEM	E_max_ ± SEM	−logEC50 ± SEM	E_max_ ± SEM	−logEC50 ± SEM	E_max_ ± SEM
**Pulmonary artery**						
Control situation	7.06 ± 0.15	83.34 ± 9.01	6.92 ± 0.11	97.64 ± 7.73	6.78 ± 0.15	95.90 ± 10.22
L-NAME	7.08 ± 0.12	141.3 ± 10.61	7.2 ± 0.13	145.2 ± 10.91	7.07 ± 0.13	141.0 ± 11.54
L-NAME + indomethacin	7.36 ± 0.10	145.7 ± 8.29	7.05 ± 0.12	126.0 ± 9.74	6.77 ± 0.21	121.3 ± 15.90
Cocktail	7.33 ± 0.08	141.3 ± 7.33	7.16 ± 0.19	121.8 ± 8.18	6.83 ± 0.18	110.8 ± 12.50

	−logEC50 ± SEM	R_max_ ± SEM	−logEC50 ± SEM	R_max_ ± SEM	−logEC50 ± SEM	R_max_ ± SEM
**Mesenteric artery**						
Control situation	6.89 ± 0.15	59.61 ± 4.70	6.96 ± 0.12	61.81 ± 3.51	7.16 ± 0.11	59.28 ± 3.47
L-NAME	6.54 ± 0.57	20.42 ± 2.92	7.12 ± 0.26	18.90 ± 1.94	6.08 ± 0.62	48.40 ± 12.84
L-NAME + indomethacin	6.95 ± 0.83	27.78 ± 4.48	7.75 ± 0.28	27.37 ± 2.80	7.29 ± 0.22	32.70 ± 3.00
Cocktail	8.045 ± 0.22	18.21 ± 1.51	7.58 ± 0.10	18.87 ± 0.82	7.39 ± 0.13	15.99 ± 0.90

After pharmacological inhibition of all major pathways for endothelium-dependent relaxation, i.e. pre-incubation with L-NAME, indomethacin, TRAM-34 and apamin, no further significant change in U46619 induced contraction was observed (Fig. 3, Table 2), suggesting that there was no significant contribution of the endothelium-dependent hyperpolarizing (EDH) component to the control of wall tension in these arteries under these experimental conditions (Supplementary fig. S4D, Table 2, *P* < 0.0001).

**Figure 4:**
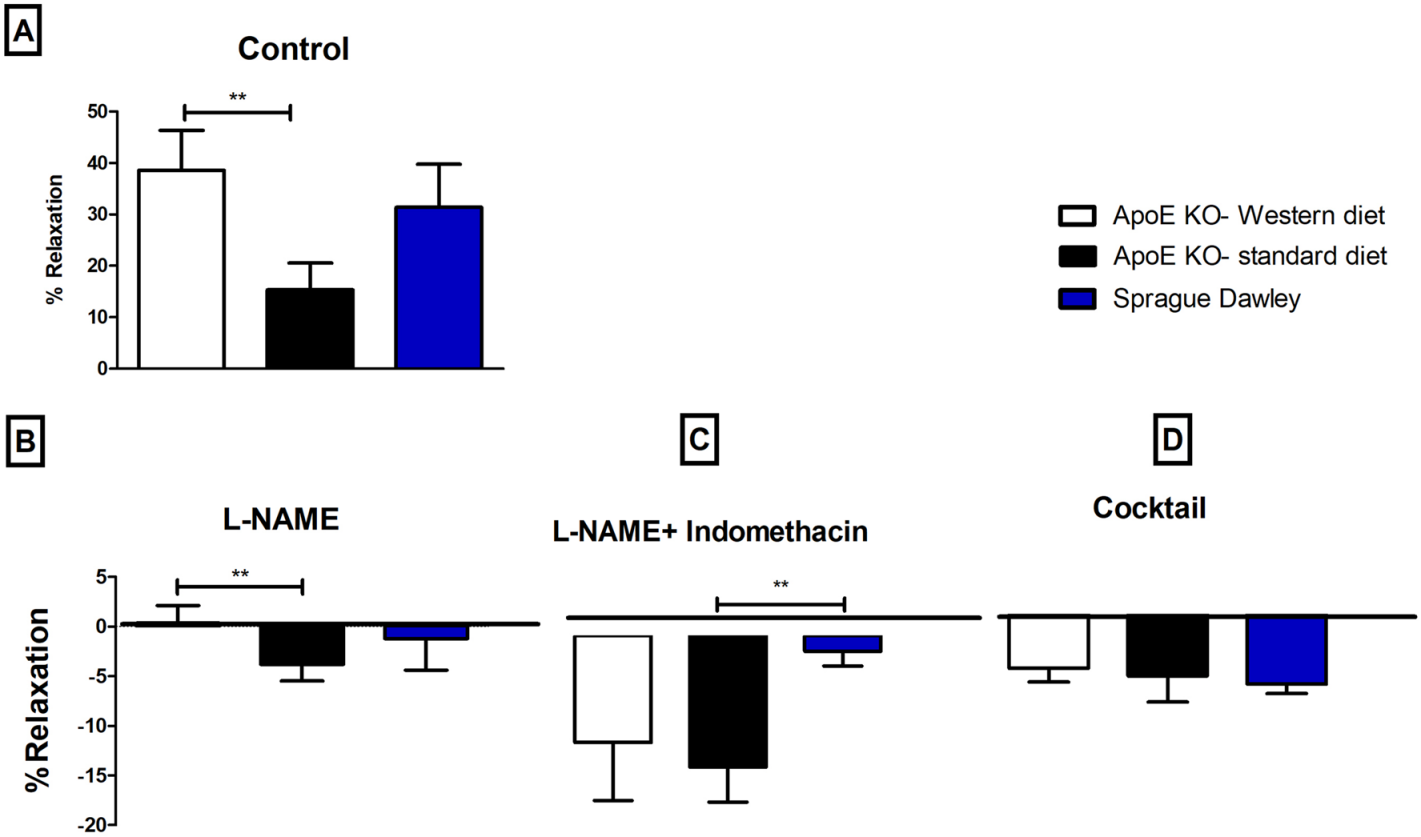
Percentage of relaxation in pulmonary arteries in response to 10–5 M concentration of ACh under different experimental conditions; (**A**) control conditions, (**), p < 0.01, ApoE KO-western diet vs ApoE KO-control diet; (**B**) incubation for 20 min with non-selective inhibitor of NO synthase, 100 µML-NAME, (**), p < 0.001, ApoE KO-Western diet vs ApoE KO-standard diet; (**C**) incubation for 20 min with both L-NAME and 3 µM indomethacin, (**), p < 0.001, ApoE KO-standard diet vs Sprague Dawley ; (**D**) 20 min incubation with Cocktail: L-NAME, indomethacin, TRAM-34 and apamin, i.e. pharmacological inhibition of all major pathways for endothelium-dependent relaxation. ANOVA, n = 8, 7.

Vessel relaxation was expressed as percentage of the pre-constricted level (0% relaxation) to passive wall tension (100% relaxation). Pre-constricted pulmonary arteries were compared also for their ACh-induced relaxation responses under control conditions. A high concentration of ACh (10^-5^ M) caused greater relaxation in pulmonary arteries from ApoE KO rats on a Western diet compared to that of a standard diet (Fig. 4A; 38.58 ± 7.75 vs 15.34 ± 5.20, *P* = 0.03, *n* = 8 and 7). However, ACh-induced relaxation was not significantly different between pulmonary arteries of Sprague Dawley rats and ApoE rats on either diet (Fig. 4A).

Relaxation was significantly suppressed in all groups after 20 min incubation with L-NAME (Fig. 4B, P < 0.001). However, the significant difference between ApoE KO-Western diet and ApoE KO-standard diet remained (Fig. 4B, 38.58 ± 7.75 vs 42.39 ± 8.46, *P* = 0.0002, *n* = 8, 7).

After adding indomethacin and L-NAME there was no relaxation in responses to ACh in any group (Fig. 4C). When all major pathways for endothelium-dependent relaxation were blocked with L-NAME, indomethacin, TRAM-34 and apamin, the relaxation was abolished in pulmonary arteries of all groups (Fig. 4D). This further supports the lack of significant contribution of the EDH signaling in these pulmonary arteries under these experimental conditions.

**Figure 5:**
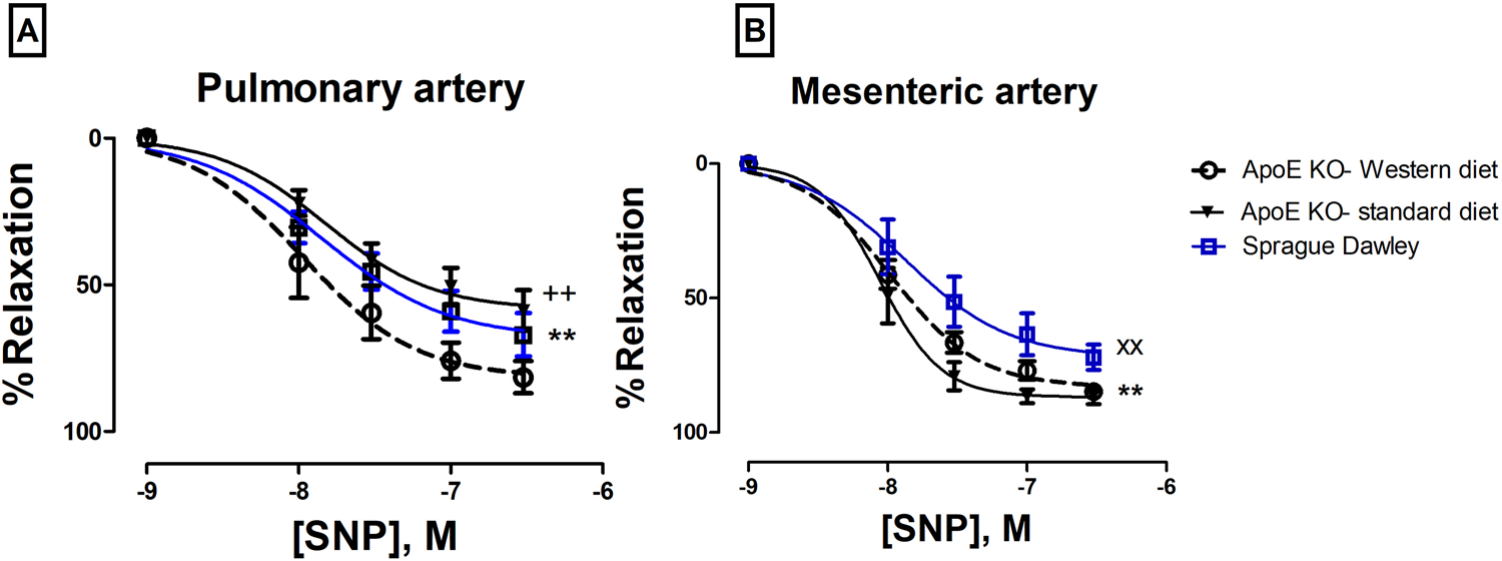
SNP concentration–response curves of (**A**) pulmonary arteries, (**), p = 0.03, ApoE KO Western diet vs Sprague Dawley, (+ +), P = 0.0002, ApoE KO Western diet vs ApoE KO-standard diet; (**B**) mesenteric arteries, (**), P = 0.02, ApoE KO Western diet vs Sprague Dawley, (xx), P = 0.0003, ApoE KO-standard diet vs Sprague Dawley; n = 8, 7.

When endothelial-independent relaxation was tested by sodium nitroprusside (SNP), there was a significant difference between the responses of arteries from ApoE KO-Western diet and either Sprague Dawley rats or ApoE KO-standard diet (Fig. 5A).

### NO was a major contributor to relaxation of the mesentery arteries in all groups

**Figure 6:**
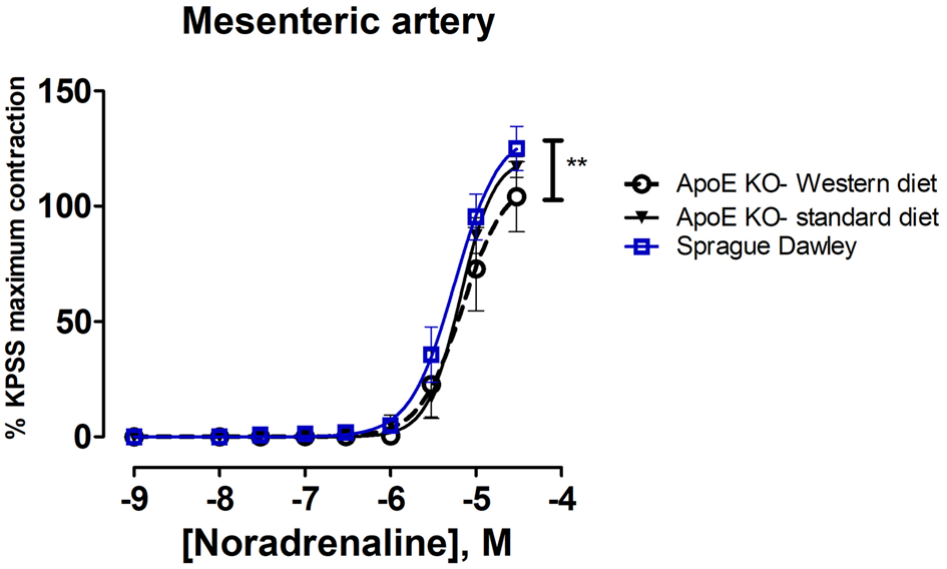
Concentration–response curves to noradrenaline of mesenteric arteries. (***), P < 0.05 ApoE KO Western diet vs Sprague Dawley rats. n = 8, n = 7.

There were no differences in contractile responses to KPSS in mesenteric arteries between the experimental groups (Supplementary fig. S3B). Contractile responses to NA in mesenteric arteries from Sprague Dawley rats were significantly larger than in the ApoE KO-Western diet group (Fig. 6, *P* = 0.01).

**Figure 7:**
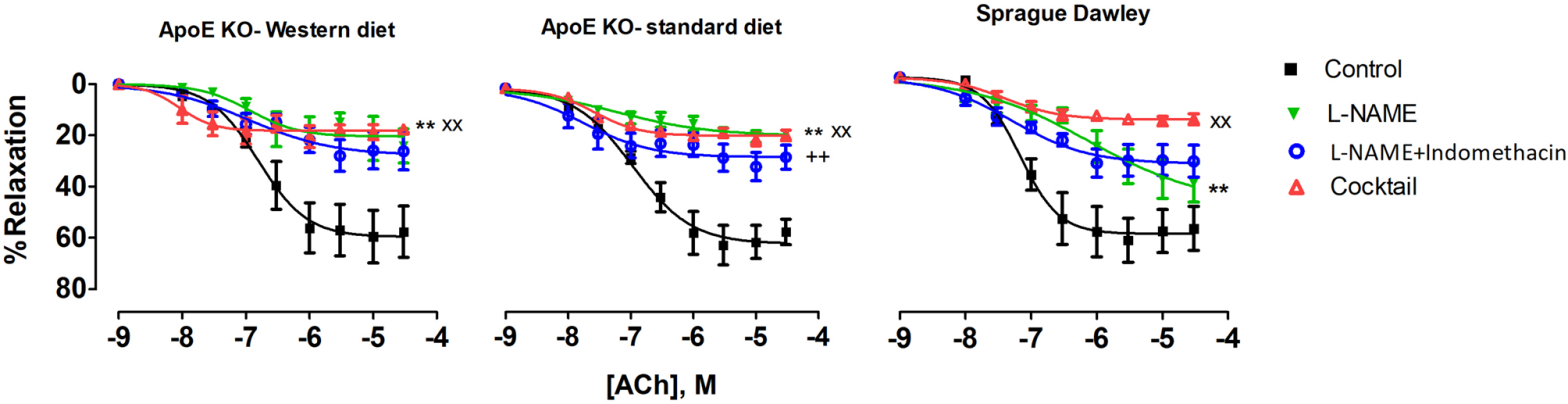
ACh concentration–response curves of mesenteric arteries from different experimental groups under; control conditions; after 20 min incubation with non-selective inhibitor of NO synthase, L-NAME (100 µM); after the incubation with both L-NAME (100 µM) and indomethacin (3 µM); after 20 min incubation with Cocktail: L-NAME, indomethacin, TRAM-34 and apamin, i.e. pharmacological inhibition of all major pathways for endothelium-dependent relaxation; (**), P < 0.001, effect of L-NAME; (+ +), P < 0.001, effect of indomethacin, (xx), P < 0.001, effect of Cocktail, n = 8, n = 7.

There were no differences in ACh-induced relaxation between experimental groups under control conditions (Supplementary fig. S5A). Incubation with L-NAME significantly suppressed the relaxation in all groups. This suppression was larger in both groups of ApoE KO rats compared to Sprague Dawley rats (Fig. 7, Table 2, *P* < 0.001), suggesting a greater contribution of NO to endothelium-dependent relaxation in mesenteric arteries from ApoE KO rats.

Incubation with indomethacin (in the presence of L-NAME) did not affected the relaxation in the ApoE KO-Western diet group (Fig. 7). This was however significantly improved in the ApoE KO-standard diet group (Fig. 7, *P* < 0.001). There was also a strong tendency for improvement (Fig. 7, *P* < 0.07) in mesenteric small arteries from Sprague Dawley rats. This suggests that in ApoE KO-standard diet and Sprague Dawley groups, product(s) of COX activity had pro-contractile effect under these experimental conditions. In the presence of L-NAME and indomethacin, no difference in ACh-induced relaxations between the groups was seen (Supplementary fig. S5C).

After pre-incubation with L-NAME, indomethacin, apamin and TRAM-34, there was significant suppression of relaxation responses in all groups (Fig. 7, Table 2, *P* < 0.001), suggesting a contribution of EDH signaling. The effect of incubation with all inhibitors was slightly but significantly different between ApoE KOs and Sprague Dawley rats on standard diet (Supplementary fig. S5, *P* < 0.002).

Endothelium-independent relaxation was tested by SNP. There was a significant difference in relaxation responses between Sprague Dawley rats and either, ApoE KO-Western diet rats (Fig. 5B; −logEC50, 7.86 ± 0.16 vs 7.97 ± 0.05, *p* = 0.02, *n* = 8) or ApoE KO-standard diet rats (Fig. 5B; −logEC50, 8.05 ± 0.06 *p* = 0.0003, *n* = 7). These results confirm our finding of increased sensitivity of smooth muscle cells to NO in arteries of ApoE KOs.

### No detectable changes in cyclooxygenase protein expression

Our functional study suggested that the Western diet modified cyclooxygenase (COX)-dependent signaling in the pulmonary artery of ApoE KO rats. However, we found no difference in COX-1 and COX-2 protein expression between the groups (Supplementary fig. S6).

## Discussion

This is the first study addressing endothelial function in arteries from ApoE KO rats fed a Western diet. The main finding was that endothelial dysfunction in male ApoE KOs on a Western diet was associated with changes in COX-dependent signaling in pulmonary arteries despite no detectable changes in the protein expression of COX-1 and -2. Moreover, we found that smooth muscle cells in mesenteric arteries of ApoE KOs were more sensitive to NO, and suggest that this might be a compensation for oxidative stress-reduced NO bioavailability resulting from hypercholesteremia. Accordingly, ApoE KO rats had significantly higher levels of plasma cholesterol, LDL and triglycerides compared to the other experimental groups. We also observed early atherosclerotic lesions, such as small foam cell lesions and accumulation of macrophages, in the aortic arch of ApoE KO rats on Western diet.

Endothelium-dependent relaxation has been reported to be impaired in atherosclerotic arteries in humans and experimental animals even before atherosclerotic lesions appear; more severely in males than in females pre-menopausal^24^. However, this sex-dependent effect in rodents is still under debate; some studies have reported that females are more susceptible, while others have reported that males are more susceptible^25,26^. In CRISPR/Cas9-generated ApoE KO rats, males developed more severe atherosclerosis than females^23^. In this current study we did not aim to understand the sex differences in atherosclerosis development in ApoE KO rats and therefore studied only males.

Small resistance arteries are affected by dyslipidemia and contribute to increases in vascular resistance^11,12^. These arteries develop endothelial dysfunction prior to atherosclerotic changes in large arteries^11,12^. In the healthy endothelium, there is a balance between vasodilator and vasoconstrictor substances, and any imbalance leads to endothelial dysfunction^27^. The endothelium produces vasodilator substances such as NO, prostaglandins (PGs), prostacyclin and EDH, as well as vasoconstrictor substances such as superoxide anion O_2_^-^, endothelin-1 (ET-1) and thromboxane A2^28,29^. The balance between these factors defines the final endothelium contribution to vascular tone.

Reduced NO bioavailability is recognized as a key factor for endothelium-dependent relaxation dysfunction in rabbits and ApoE/LDL receptor-knockout mice fed a high fat diet^24,30^. NO is the most important vasodilator in both resistance and large arteries^10^. Increased reactive oxygen species (ROS), especially O_2_^-^ as a result of hypercholesterolemia, could affect the bioavailability of NO^31–33^. To investigate the role of NO in endothelium-dependent relaxation, we incubated pulmonary arteries with L-NAME (non-selective inhibitor of NO synthase). However, no differences in L-NAME effect on ACh-induced relaxation or U46619-induced contraction between ApoE KOs on Western diet and ApoE KOs or Sprague Dawley rats on standard diet were observed. The role of hypocholesteremia in excess ROS production and oxidative stress has been shown before^33^. In a previous study on ApoE KO rats, we observed no increase in oxidative stress in the pulmonary artery of ApoE KO rats when assessed by lipid peroxidation in plasma. Moreover, we found that Tempol, a superoxide scavenger, did not affect endothelium-dependent relaxation^34^. However, in the previous study the rats were younger (6–9 weeks old) and fed a standard diet while in the current study, ApoE KO rats were fed a Western diet and developed significant hypercholesterolemia. However, the L-NAME sensitive component of endothelium-dependent relaxation was still unchanged. In this term, it is surprising that the sensitivity to NO assessed with the endothelium-independent SNP relaxation was increased in ApoE KO rats on the Western diet. We suggest therefore that NO bioavailability might be reduced in pulmonary arteries from these ApoE KO rats on Western diet, possibly because of dyslipidaemia-induced oxidative stress, which may have been compensated by increased sensitivity of smooth muscle cells to NO. Further studies with direct measurements of NO and cGMP-dependent signaling in smooth muscles are necessary to clarify this possibility.

In cardiovascular disease associated with atherosclerosis, there is a shift in the balance of the different endothelium-derived factors. Importantly, there is increased production of endothelium-derived contractile factors; this occurs independently of reduced NO production or availability and is also associated with increased oxidative stress^35,36^. Oxidative stress has been shown to trigger endothelium-derived contraction by upregulation of COX-1 and/or the induction of COX-2^10,37^. In this study, we found no relaxation in ApoE KO-Western diet rats after incubation of the pulmonary artery with both L-NAME and indomethacin, a non-selective inhibitor of COX enzymes. This is in accordance to previous findings in renal arteries of male Wistar-Kyoto rats, suggesting the key importance of NO and COX associated pathways of endothelium-dependent relaxation^38^. We did not, however, observe any changes in protein expression of COX-1 and COX-2 in ApoE KOs, either on Western or standard diet, therefore the COX-dependent contraction was apparently not related to expressional changes of COXs enzymes but is possibly due to changes in the activity of downstream enzymes or receptor distribution.

COX1 and COX2 are important in the regulation of vascular tone. These enzymes are involved in the formation of prostanoids such as prostacyclin (PGI_2_), a vasorelaxant factor, and thromboxane A2, isoprostanes and PGH_2_, which are vasoconstrictors^38–40^. There is a balance between these COX products under normal conditions, but this can change with aging or disease^10^. Pro-contractile prostanoids and thromboxane A2 are increased in atherosclerosis and account for most of the endothelium-mediated contraction^39,41^. COX-1 is the main source of thromboxane A2 and COX-1 deletion prevents atherosclerotic lesion formation in ApoE null mice^39^. On the other hand, aging and hypertension suppresses prostacyclin-induced arterial relaxation^27,35,41^. Studies of spontaneously hypertensive rats (SHR) and WKY rats have shown that ACh causes a considerable release of prostacyclin. However, the ability of prostacyclin to induce relaxation decreases in aged and hypertensive rats; instead it elicits contraction^35^. This can explain the results of the present study, where despite of unchanged COX expression, we observed functional changes of indomethacin that were different between the groups. Our findings suggest that small arteries from ApoE KO rats develop endothelial dysfunction prior to the development of atherosclerotic lesions^39,41^. Interestingly, our study shows that in rat pulmonary arteries, COX activity produced pro-contractile net-effect in Sprague Dawley rats and ApoE KO rats fed the standard diet, while vasorelaxing COX action was only seen in ApoE KO rats fed a Western diet. Whether this is an adaptive change, for example for putative reduction in NO bioavailability or production as discussed above, or a result of pathological development due to hypercholesterolemia remains to be shown. Importantly, U46619 is a thromboxane A_2_ agonist and this can further complicate the results.

The responses of mesenteric arteries from ApoE KO and Sprague Dawley rats were different from that of the pulmonary arteries. Given that L-NAME strongly suppressed ACh-induced relaxation, it could be concluded that NO had a significant role in endothelium-dependent relaxation in ApoE KO mesenteric arteries, irrespective of diet. It was previously shown that NO-mediated endothelium-dependent relaxation is impaired in ApoE KO mice fed a Western diet due to increased O_2_^-^ production^33^. Similar results were observed in aorta of 32–35 months-old Sprague Dawley rats despite increased NO synthase expression and increased activity of the l-arginine/NO pathway^42^. In atherosclerosis prone arteries, the increased O_2_^-^ has a significant role in deactivating NO and forming peroxynitrite^33,42^. This seems to be the case in our study. Stronger suppression of endothelium-dependent relaxation of mesenteric arteries from ApoE KO rats in the presence of L-NAME suggests larger NO signaling that might be a mechanism to compensate the increased production of O_2_^-^ as a result of hypercholesteremia. Since we did not directly measure NO production and its bioavailability, we cannot specify the changes in NO signaling. However, an increased NO sensitivity seen in the experiment with SNP suggests that this potentiation of NO signaling might be at the level of smooth muscle cells.

A small effect was observed after inhibition of both COX and EDH signaling in mesentery arteries from ApoE KO rats on Western diet. This suggests that EDH signaling was suppressed in this experimental group in comparison with Sprague Dawley rats. This is in accordance with previous observations in mesenteric arteries of diabetic rats^43^. In contrast, COX inhibition slightly improved the relaxation of the arteries from ApoE KO rats on the standard diet, suggesting some pro-contractile action of COX products in these arteries. EDH inhibition suppressed this relaxation, indicating that some vasorelaxing EDH effect was present in the mesenteric arteries from ApoE KO rats on the standard diet but was suppressed after exposure to the Western diet. However, the contribution of EDH signaling was clearly reduced in ApoE KO rats on any diet in comparison with Sprague Dawley rats, although Sprague Dawley rats did not show any significant COX-dependent response. This is in accordance with previous reports suggesting that COX signaling is not significant for rat mesenteric arteries under normal condition^44^. Thus, mesenteric arteries from ApoE KO rats show an increased contribution of NO in comparison with Sprague Dawley rats, and this is balanced by an increased pro-contractile action of COX products in ApoE KO rats on the standard diet and by diminished EDH signaling in those on the Western diet.

Though providing highly standardized experimental conditions, pharmacological studies of endothelial function using the isometric myograph has significant limitations. We used ACh to induce endothelium-dependent responses, while in in vivo conditions endothelium-dependent factors modulate vascular tone under influence of blood flow changes and numerous humoral factors. We do not know whether hemodynamic parameters in vivo were different between the groups of rats. It is also possible that dyslipidaemia and associated inflammation could contribute to the endothelial state in ApoE KO rats in vivo while these humoral factors were not present in the myograph. These uncertainties should be considered in future studies. In the present study, only ApoE KO rats were fed the Western diet. However, this diet itself has been shown to induce the formation of reactive oxygen species and inflammation^45,46^, although this is not in agreement with other studies^16^. It is therefore possible that some Western diet associated changes observed in our ApoE KO rats, would also be seen in Sprague Dawley rats if they were fed the same diet. This was however out of the scope of our study into whether Western diet can accelerate endothelial dysfunction and earlier atherosclerosis in ApoE KO rats specifically.

## Conclusion

To our knowledge this is the first report examining the vascular phenotype in ApoE KO rats fed a Western diet. The results are in accordance with previous data on atherosclerosis obtained in other models of atherosclerosis, in which changes in COX contribution to vascular tone is associated with endothelial dysfunction at early stage of atherosclerosis and the significant role of oxidative stress^32,41,42^. An animal model for atherosclerosis that better mimics the time course of the disease in humans will improve our understanding of the therapeutic utility of COX inhibitors^40,47^. The ApoE KO rats are a new model to study endothelial dysfunction during the earlier stages of atherosclerosis and could help us improve preclinical drug development.

## Methods

A summary chart of the study design is shown in supplementary fig. S1.

### Animals

15 male ApoE KO and 8 male Sprague Dawley rats aged 11 to 13 weeks old were used (Horizon Discovery, Saint Louis, USA). The Zinc Finger (ZF) technique was used by Horizon Discovery to produce the ApoE KO rats. Animals were housed 2 per cage (temperature 21 ± 1 °C, 12–12 h light–dark cycle). ApoE KO rats were randomly subjected to either a Western diet (weight 360.10 ± 8.39 g, *n* = 8) or a standard diet (weight 352.10 ± 7.94 g, *n* = 7), where 41% and 10% of the energy comes from fat, respectively (Cat. #D12079B and Cat. #D 98,121,701, Brogaarden Korn & Foder ApS, Lynge, Denmark). Sprague Dawley rats were included as a genetic background control^22^ and fed the standard diet (weight, 373.9 ± 7.69, *n* = 8). All animals had ad libitum access to food and water. The project continued for 20–24 weeks. After 20–24 weeks of Western or standard diet, rats were anesthetized with Sevofloran (Sevorane, AbbVie, Copenhagen, Denmark) and euthanized by decapitation at age of 31–37 weeks old.

### Isometric force measurement

The pulmonary artery (first bronchial artery in right lobe) and third order mesenteric were dissected and mounted in an isometric myograph (Danish Myo Technology, Aarhus, Denmark) as described previously^34^. Maximal contractile response was assessed with potassium solution (KPSS): equimolar substitution of KCl for NaCl in PSS. Contractility of pulmonary and mesenteric arteries were tested by cumulative applications of the thromboxane A_2_ receptor agonist, U46619 (10^−8^–3×10^−6^ M), and noradrenaline (NA, 10^−8^–3×10^−5^ M). Endothelial function was assessed by relaxing pre-constricted arteries with acetylcholine (ACh: 10^–7^, 10^–6^ and 10^–5^ M). Pre-constrictions to approximately 70% of the KPSS-induced maximal constriction were obtained with either U46619 or NA-mediated stimulations of the pulmonary and mesenteric arteries, respectively. No time effect was observed in separate time-control experiments (Supplementary fig. S2).

### Blood sampling and tissue collection

Blood samples from the tail vein were collected in 500 ul EDTA tubes (Microvette 500 K3E, Hounisen, Skanderborg, Denmark) monthly from the onset of the diet while the rats were under sevoflurane anesthesia. The last sample was collected from the left heart ventricle in 4 mL EDTA vacutainer tubes immediately following anesthesia after the rats had fasted overnight. All blood samples were centrifuged at 4.000 g for 10 min at 4 °C within 30 min of collection. Aspirated plasma was stored at − 80 °C until assayed. The rest of the pulmonary arteries that did not go into the functional studies were dissected and snap-frozen in liquid N_2_ and stored at − 80 °C until analysis.

### Immunohistochemistry

The aortic arch and thoracic aorta were isolated and fixed in 4% PFA (Paraformaldehyde, Alfa Aesar, USA) at 4 ºC overnight, and then embedded in paraffin. Tissues Sects. (3–4 µm) were mounted on slides and kept at room temperature until staining. Sections were deparaffinized and stained with hematoxylin and eosin (H & E, Sigma-Aldrich, Oslo, Norway) and rabbit polyclonal CD68 (CD-68, Abcam, Cambridge, UK). Staining was imaged under a microscope as previously described^23,48^.

### Blood chemistry

The total cholesterol, HDL and triglyceride contents of the plasma samples were analyzed using an enzymatic colorimetric method (Roche, Basel, Switzerland) on the Cobas C111 system (Roche Diagnostics, Basal, Switzerland).

### Western blot

Pulmonary arteries collected from 22 rats were used in Western blot analysis, one sample was missing due to technical issues. Total protein content in pulmonary arteries was quantified using the bicinchoninic acid (BCA) protein assay kit (Thermo Scientific, Massachusetts, USA). Samples were measured at 562 nm (PHERAstar, BMG Labtech, Ortenberg, Germany) after 30 min incubation at 37 °C. The supernatant was used to prepare gels while in Laemmli sample buffer containing 2% SDS. 12% Criterion Precast Gel (Bio-Rad laboratories, Copenhagen, Denmark) was used to separate proteins, which were then electrotransferred to a nitrocellulose membrane. Blots were blocked with 5% nonfat dry milk in PBS-T as described previously^49^. After washing with PBS-T, blots were incubated with primary antibodies (COX-1, COX-2 and beta-actin) overnight at 4 °C, and visualized with horseradish peroxidase (HRP)-conjugated secondary antibodies for 1 h at room temperature using the enhanced chemiluminescence system (ECL Plus, GE Healthcare, Amersham, United Kingdom). Values were normalized to total protein using Stain-Free technology.

### Data analyses

GraphPad Prism software (5.02 for Windows, LaJolla, California, USA) was used for data analysis, similar to previous studies^34^. The effects of inhibitors were calculated as a comparison of difference in concentration–response curves before and after administration of the drug. From these curves, –logEC_50_, where EC_50_ was the concentration required to produce a half-maximal response, and maximal response were derived and compared using an extra sum-of-squares *F* test. Results are presented as means ± SEM. Differences between means were tested by one-way ANOVA followed by Bonferroni post hoc-test or by t-test statistics and the results presented as means ± SD (standard deviation of the mean). *P* < 0.05 was considered statistically significant.

### Ethical statement

Experiments were approved by Ministry of Environment and Food of Denmark for animal experiments, approval number 2018–15-0,201–01,477. All studies were performed in accordance with Animal Research: Reporting of In Vivo Experiments (ARRIVE) and the European Convention for the Protection of Vertebrate Animals used for experimental and other scientific purposes.

## Supplementary information

Supplementary Information
