# Supplementary material for: Endothelial dysfunction in small arteries and early signs of atherosclerosis in ApoE knockout rats
Source: Sci Rep. 2020 Sep 17;10:15296. doi: 10.1038/s41598-020-72338-3 (PMC7499202; doi:10.1038/s41598-020-72338-3)
Supplement: Supplementary file 1 — Supplementary Information [file 41598_2020_72338_MOESM1_ESM.pdf]

Phenotyping ApoE knockout rats on a Western diet: endothelial dysfunction in small arteries as early signs of atherosclerosis

Simin Berenji Ardestani[1,2], Ingrid Eftedal[2,3], Michael Pedersen[1], Per Bendix Jeppesen[1], Rikke Nørregaard[1], Vladimir V. Matchkov[4]

[1] Department of Clinical Medicine, Aarhus University, Denmark

[2] Department of Circulation and Medical Imaging, Faculty of Medicine and Health Sciences, NTNU Norwegian University of Science and Technology, Trondheim, Norway.

[3] Faculty of nursing and health sciences, Nord University, Bodø, Norway

[4] Department of Biomedicine, MEMBRANES, Health, Aarhus University, Aarhus, Denmark

Corresponding author

Simin Berenji Ardestani, Department of Clinical Medicine,

Aarhus University,

Palle Juul Jensens Boulevard 99,

DK8200 Aarhus N, Denmark

Email: Simin.berenji@clin.au.dk

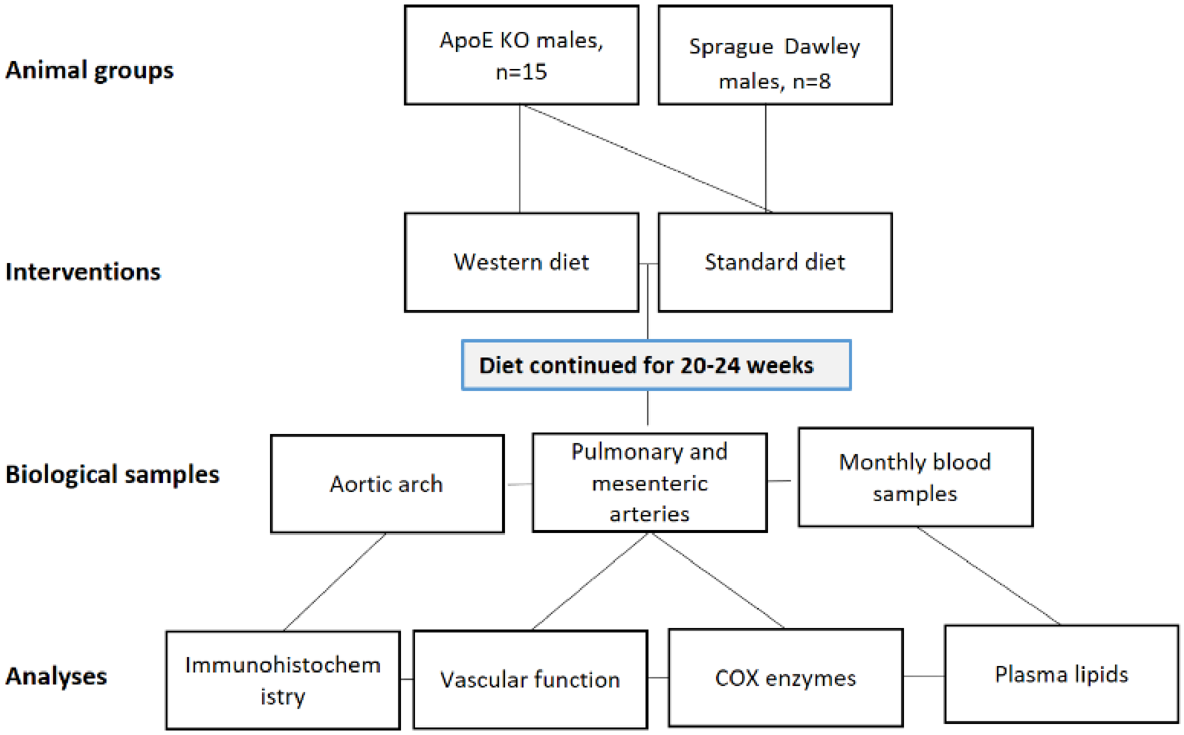

Supplementary Figure S1. A summery chart of study design. 15 male ApoE knockout (KO) rats and 8 Sprague Dawley rats were subjected to either a Western or standard diet for 20-24 weeks

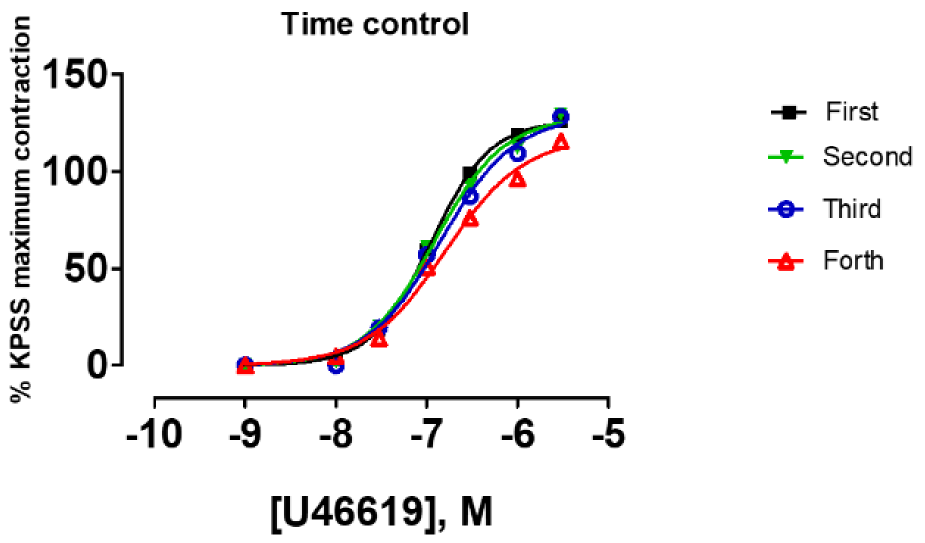

Supplementary Figure S2. Time control in pulmonary arteries. Percentage of concentration, expressed relative to the maximal contraction of KPSS, in response to U46619 in pulmonary arteries

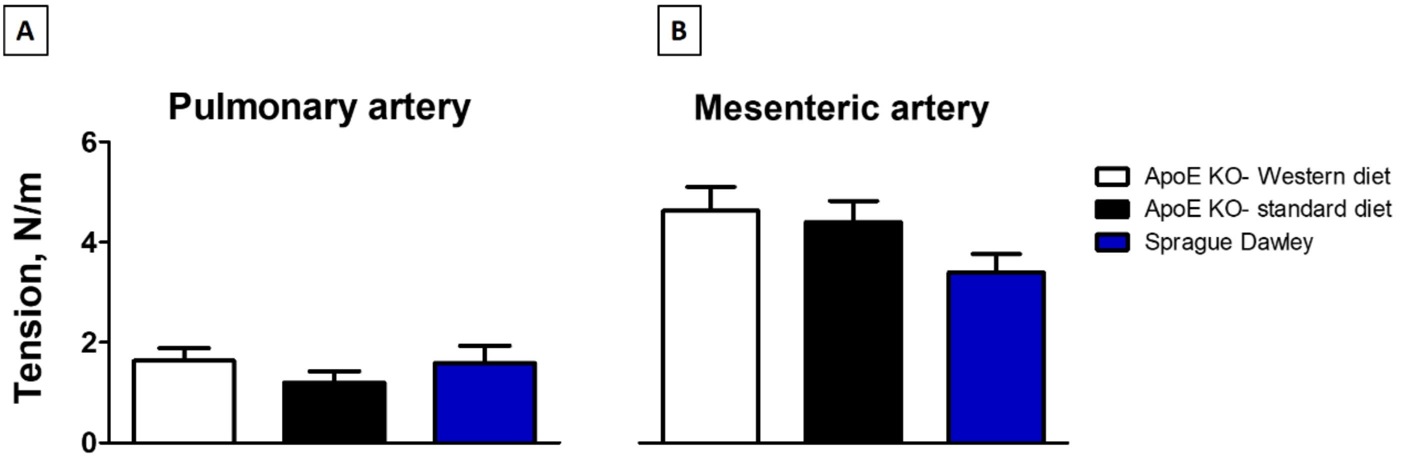

Supplementary Figure S3. Maximal contraction in presence of KPSS. (A) pulmonary (B) mesenteric arteries. KPSS, potassium physiological saline solution ANOVA, n=8, n=7.

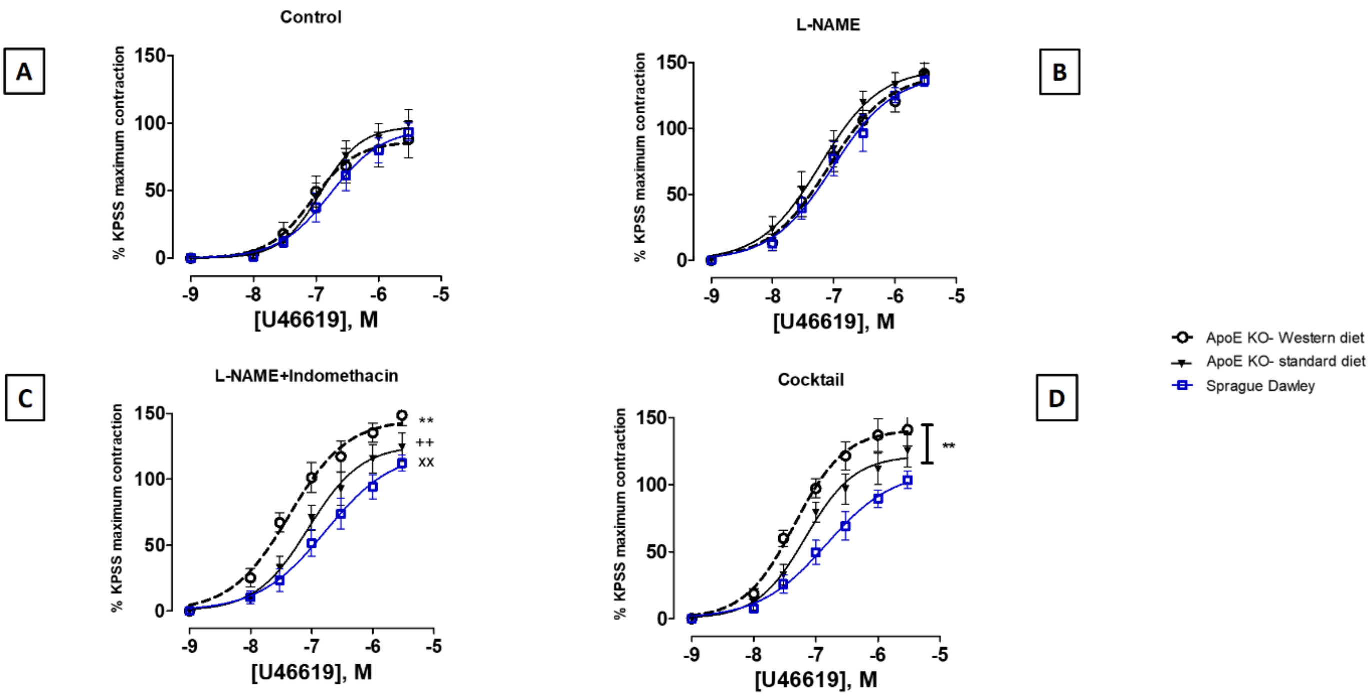

Supplementary Figure S4. U46619 concentration-response curves of pulmonary arteries from different experimental groups under different conditions; (A), Control conditions; (B), In the presence L-NAME (100 µM); (C), After 20 min incubation with L-NAME (100 µM) and indomethacin (3 µM), (**), P<0.0001 for ApoE KO- Western diet vs ApoE- control diet, (++), P=0.09, ApoE KO- control diet vs Sprague Dawley, (xx), P<0.0001, Sprague Dawley vs ApoE KO- Western diet; (D), The effect of 20 min incubation with Cocktail: L-NAME, indomethacin, TRAM-34 and apamin, i.e. pharmacological inhibition of all major pathways for endothelium-dependent relaxation, (**), p=0.001, ApoE KO- western diet vs ApoE Ko- control diet, (++), p<0.001, ApoE KO- standard diet vs Sprague Dawley, (xx), P<0.0001, Sprague Dawley vs ApoE KO- Western diet, F test, n=8, 7.

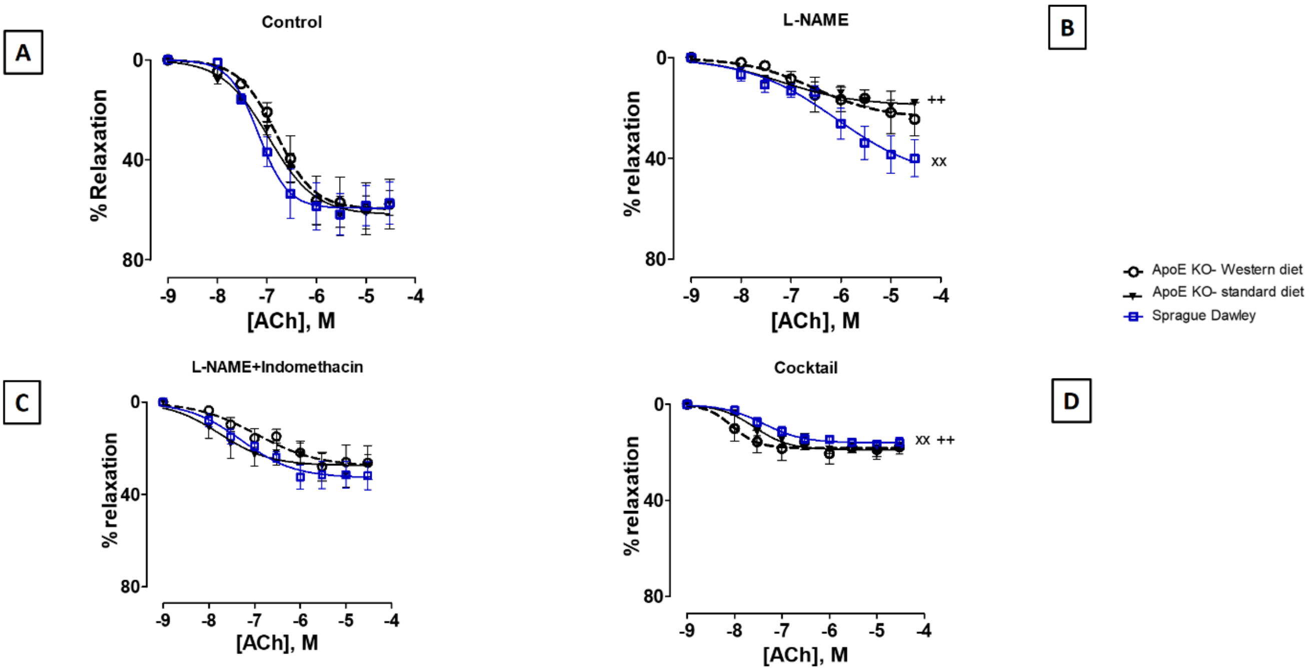

Supplementary Figure S5. concentration-response curves of mesenteric arteries from different experimental groups under different conditions; (A), Control conditions; (B), In the presence L-NAME (100 µM), (++), P<0.001, ApoE KO- control diet vs Sprague Dawley (xx), P=0.003, Sprague Dawley vs ApoE KO Western diet; (C), After 20 min incubation with L-NAME (100 µM) and indomethacin (3 µM); (D), The effect of 20 min incubation with Cocktail: L-NAME, indomethacin, TRAM-34 and apamin, i.e. pharmacological inhibition of all major pathways for endothelium-dependent relaxation, (++), p<0.002, ApoE KO standard diet vs Sprague Dawley, (xx), P<0.002, Sprague Dawley vs ApoE KO Western diet, n=8, n=7.

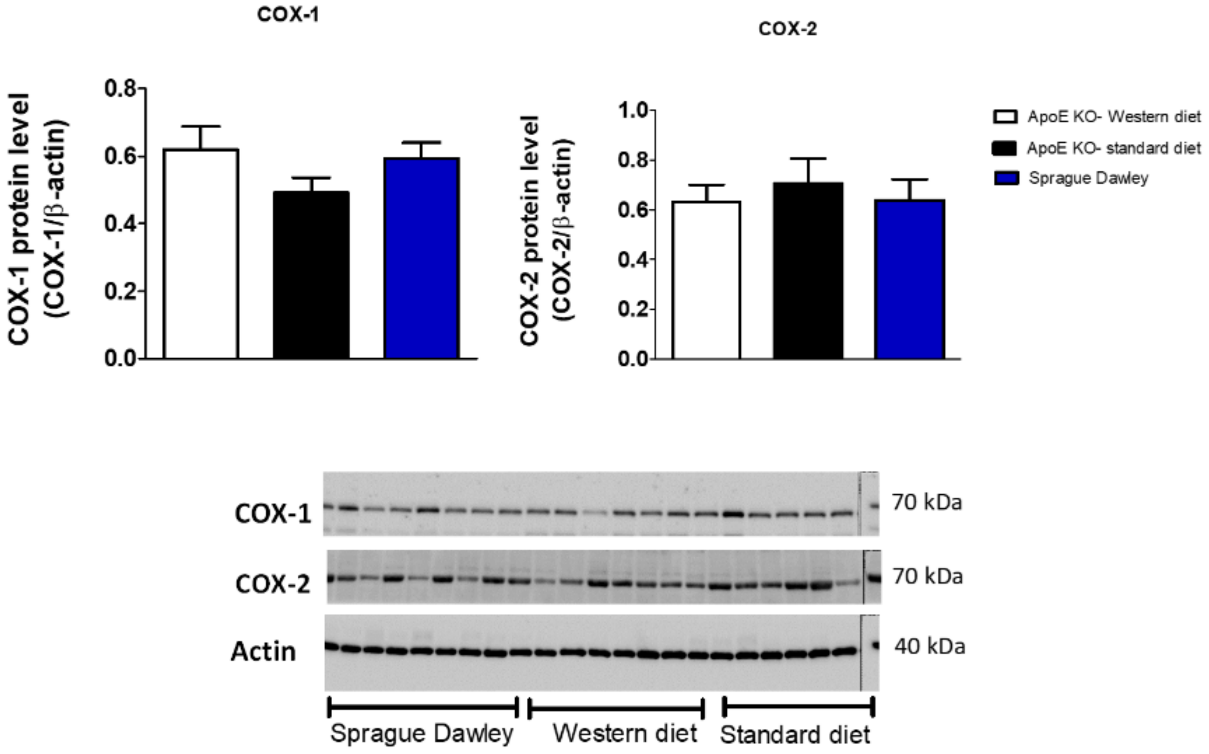

Supplementary Figure S6. Relative expression of COX1 and COX 2. Proteins quantified by Western blot and normalized to β-actin as loading control. ANOVA and t-test, n=8 ,7.
